# Flex-Printed Ear-EEG Sensors for Adequate Sleep Staging at Home

**DOI:** 10.3389/fdgth.2021.688122

**Published:** 2021-06-30

**Authors:** Carlos F. da Silva Souto, Wiebke Pätzold, Karen Insa Wolf, Marina Paul, Ida Matthiesen, Martin G. Bleichner, Stefan Debener

**Affiliations:** ^1^Branch for Hearing, Speech and Audio Technology HSA, Fraunhofer Institute for Digital Media Technology IDMT, Oldenburg, Germany; ^2^PSG-Auswertungs-Service, Stadtlohn, Germany; ^3^Neuropsychology Lab, Department of Psychology, University of Oldenburg, Oldenburg, Germany; ^4^Neurophysiology of Everyday Life Group, Department of Psychology, University of Oldenburg, Oldenburg, Germany

**Keywords:** EEG, sleep staging, grapho-elements, ear-EEG, flexible PCB electrodes, mobile EEG

## Abstract

A comfortable, discrete and robust recording of the sleep EEG signal at home is a desirable goal but has been difficult to achieve. We investigate how well flex-printed electrodes are suitable for sleep monitoring tasks in a smartphone-based home environment. The cEEGrid ear-EEG sensor has already been tested in the laboratory for measuring night sleep. Here, 10 participants slept at home and were equipped with a cEEGrid and a portable amplifier (mBrainTrain, Serbia). In addition, the EEG of Fpz, EOG_L and EOG_R was recorded. All signals were recorded wirelessly with a smartphone. On average, each participant provided data for *M* = 7.48 h. An expert sleep scorer created hypnograms and annotated grapho-elements according to AASM based on the EEG of Fpz, EOG_L and EOG_R twice, which served as the baseline agreement for further comparisons. The expert scorer also created hypnograms using bipolar channels based on combinations of cEEGrid channels only, and bipolar cEEGrid channels complemented by EOG channels. A comparison of the hypnograms based on frontal electrodes with the ones based on cEEGrid electrodes (κ = 0.67) and the ones based on cEEGrid complemented by EOG channels (κ = 0.75) both showed a substantial agreement, with the combination including EOG channels showing a significantly better outcome than the one without (*p* = 0.006). Moreover, signal excerpts of the conventional channels containing grapho-elements were correlated with those of the cEEGrid in order to determine the cEEGrid channel combination that optimally represents the annotated grapho-elements. The results show that the grapho-elements were well-represented by the front-facing electrode combinations. The correlation analysis of the grapho-elements resulted in an average correlation coefficient of 0.65 for the most suitable electrode configuration of the cEEGrid. The results confirm that sleep stages can be identified with electrodes placement around the ear. This opens up opportunities for miniaturized ear-EEG systems that may be self-applied by users.

## Introduction

Capturing sleep stages in a medically meaningful way requires the infrastructure of a sleep laboratory and the placement of many sensors by well-trained personnel. Acquisition of electroencephalogram (EEG) data is the most fundamental part of a polysomnography (PSG). While the PSG administered in the sleep lab in controlled conditions continues to play an important role in sleep medicine, it has become clear that more options are needed to address different questions concerning sleep and sleep disorders. The German Society for Sleep Research and Sleep Medicine (DGSM) has long been drawing attention to the need for research with regard to simplified procedures to support sleep diagnostics with possible applications for at-home studies (1), as the ever-increasing demand for diagnostics leads to long waiting lists for sleep laboratories. Additionally, standard PSG in a sleep lab is obtrusive and may lead to atypical sleep patterns in a patient, which can complicate the diagnostic process (2).

The consumer market has identified those experiencing trouble sleeping as customers. Many gadgets, devices, and apps are available and promise information on sleep duration, sleep quality or even sleep apnea. Yet, sleep researchers and practitioners alike questioned the validity of consumer-level sleep tracking devices (3, 4). Most solutions are neither medical devices nor validated against standard procedures. Nevertheless, the marketing claims can be ambiguous, and customers may perceive the products as scientifically validated because of their appearance. Inaccurate feedback on one's sleep, however, can corrupt people's perception of their quality of sleep, worsening symptoms and hindering appropriate diagnosis and treatment. Negative feedback on sleep quality can harm daytime functioning and increase reported daytime fatigue, as tested in a sham experiment with insomniac patients (5). The development of accurate, low-threshold sleep monitoring solutions that could be self-applied and used at home may help to avoid those problems.

Several research groups and for-profit companies have developed compact EEG sleep monitoring systems that may help to re-define how sleep EEG can be taken from the scalp in ways that are easy to apply without preparation and not disrupting to wear during sleep.They differ in placement (face, ear) as well as type and number of channels but have in common that they present ideas outside of the box of standard PSG, with some reporting promising results. Recent examples for facial solutions include self-applicable electrodes developed for emergency medicine (6, 7), a printed dry-electrode array applied to the face (8) and auto-adhesive electrodes attached with a headband (9). Devices focusing on the ear canal include both dry and wet in-ear electrodes that fit into a personalized earpiece (10, 11) as well as in-ear sensors attached to a foam earplug (12). In the consumer market, several commercially available devices have been scientifically evaluated. Examples include sensor systems with single-use electrodes applied to the forehead (13, 14) or dry electrodes in a headband, with brush-like silicon electrodes at the back of the head and flat dry electrodes on the forehead (15, 16). These systems have in common that they offer all necessary hardware in a compact, easy-to-use setup. However, in many cases, data analysis runs on company-owned servers, and direct access to raw data is refused, which is incompatible with independent scientific evaluation attempts.

In addition to the development of novel devices, a top-down approach to EEG may help identify simple electrode constellations that yield the best results. Databases of clinical PSG data have been analyzed to find the smallest working combination of sensors that allow solid sleep staging. Examples include evaluating sleep stages from a single electrode (17, 18) as well as the use of machine learning approaches that use the smallest number necessary for a correct categorization from a large number of available parameters and thus provide information about possible reduced sensor constellations (19, 20).

Currently, we know of no miniaturized sleep monitoring system that is fully self-applicable for the use at home in the sense that all equipment would be easily accessible to a layperson. In this study, we focused on combining the signal quality of wet scalp electrodes with the usability of easily applied dry electrodes by applying wet flex-printed electrodes to the hairless skin around the ear. By doing so, we built on existing knowledge concerning ear-EEG and get one step closer toward self-administered home sleep EEG acquisition. We tested an approach to at-home sleep monitoring, applying novel, unobtrusive hardware and using statistical means to validate the data quality and sensor selection. We focused on easily obtainable sensor parts to build a sensor system that can easily be adapted or replicated. For the EEG, the cEEGrid was used (21, 22). The cEEGrid is a flexible, discrete wet EEG system and is particularly suitable for measuring EEG in the home environment. It consists of a printed circuit board (PCB) including flat silver electrodes on a flexible polymer sheet in the shape of a C. Fitting around the hairless skin around the ear, it is self-adhesive and easy to apply, making it easy to use for measuring sleep at home.

Compared with ear-canal-electrodes or single electrodes, the cEEGrid has the advantage of larger inter-electrode distances, which allows for the recording of larger amplitude signals (23). In total, the cEEGrid offers eight channels per ear that are referenced to the mastoid. The suitability for the use of the cEEGrid for measurements during sleep for up to 12 h has already been empirically proven (11, 24). Both publications referred to the same dataset recorded data in a sleep laboratory and employed cEEGrids on two ears (24), found that sleep stages were difficult to differentiate based on cEEGrid channels compared to a full PSG, while differentiation between sleep and wake showed slightly higher agreement (25) found that the automatic scoring of cEEGrid data lead to a similar accuracy as the expert scoring of a standard PSG, which encourages the further exploration of cEEGrid sleep data. The higher accuracy based on automatic scoring compared to manual scoring of cEEGrid data was explained due to the non-standardized positions of cEEGrid electrodes and possible inexperience in manual scoring these kinds of data. A correlation index was derived between cEEGrid and scalp derivations within specific frequency bands (alpha, beta, theta and delta) resulting in good correlations using electrode averages and larger electrode distances.

In the current study, we used a single cEEGrid, thereby reducing preparation time to very few minutes, which is one important feature when getting closer to a future self-application system. In the manual scoring of the data, linear combinations of cEEGrid channels were used and labeled accordingly to extrapolate to classical PSG channels. In addition, participants slept at home and started and finished the recording by themselves. Since home application makes the full PSG setup impractical, an approximation to relevant EEG and EOG (electrooculogram) channels of the PSG was used. Previous research showed that a single channel (Fpz) can be sufficient to differentiate REM and deep sleep stages (17). EOG channels give additional information on eye movements that facilitate sleep staging and offer essential information for sleep coders. Therefore, we measured full nights of sleep at home using one cEEGrid, one EEG channel (Fpz) and two EOG channels in a lightweight, mobile setup. Our aim was to identify sleep stages with this simple setup, enlisting a trained sleep scorer to manually code the EEG data.

To determine the reliability of the sleep expert scorer, we calculated the test-retest reliability of hypnograms that were created at two different time points (2018, 2020) using the EEG of Fpz, EOG_L and EOG_R. The results represented the best possible score for further comparisons. We then created bipolar channels based on combinations of cEEGrid channels to approximate channels traditionally used for EEG in a PSG, therefore considering the spatial filter quality of EEG channels. We then defined the cEEGrid data as the first experimental dataset, and the cEEGrid + EOG data as a second experimental dataset. Both datasets were independently coded by the sleep expert scorer according to AASM criteria for sleep staging. Hypnograms were compared to the frontal channel + EOG hypnograms. Further, we tested how similar so-called grapho-elements of the sleep EEG are represented using this setup compared to Fpz signal. We determined K-complexes, and sleep spindles, characteristic of stage two sleep as suitable grapho-elements to include in the analysis. We tested which cEEGrid channel combinations would give the best representation of the annotated grapho-elements. Therefore, K-complexes and sleep spindles annotated by the expert scorer in the Fpz-EOG were correlated with every channel and all possible channel pair combinations of the cEEGrid-EEG. This correlation analysis explored whether combinations of ear EEG channels can be used to mimic the EEG measured at further distanced location of the scalp, like Fpz. If supported, this approach would motivate a selection of cEEGrid channel combinations which could approximately represent the measured EEG of classical PSG scalp electrodes like Fp2, F4, C4, P4 and O2.

## Materials and Methods

### Participants

Ten participants (8 females, 2 males, mean age = 28.4 ± 4.3 years) were recruited from members and friends of the Department of Psychology, Carl von Ossietzky University of Oldenburg, Germany. Recruiting among this group was necessary because participants were visited by the experimenter in their private house and it was preferred that she is familiar to the participants. Participants reported no sleep disorders. Each participant provided one night of sleep data, recorded during sleep at home. The study was approved by the local ethics board.

### Data Acquisition

**Figure 1:**
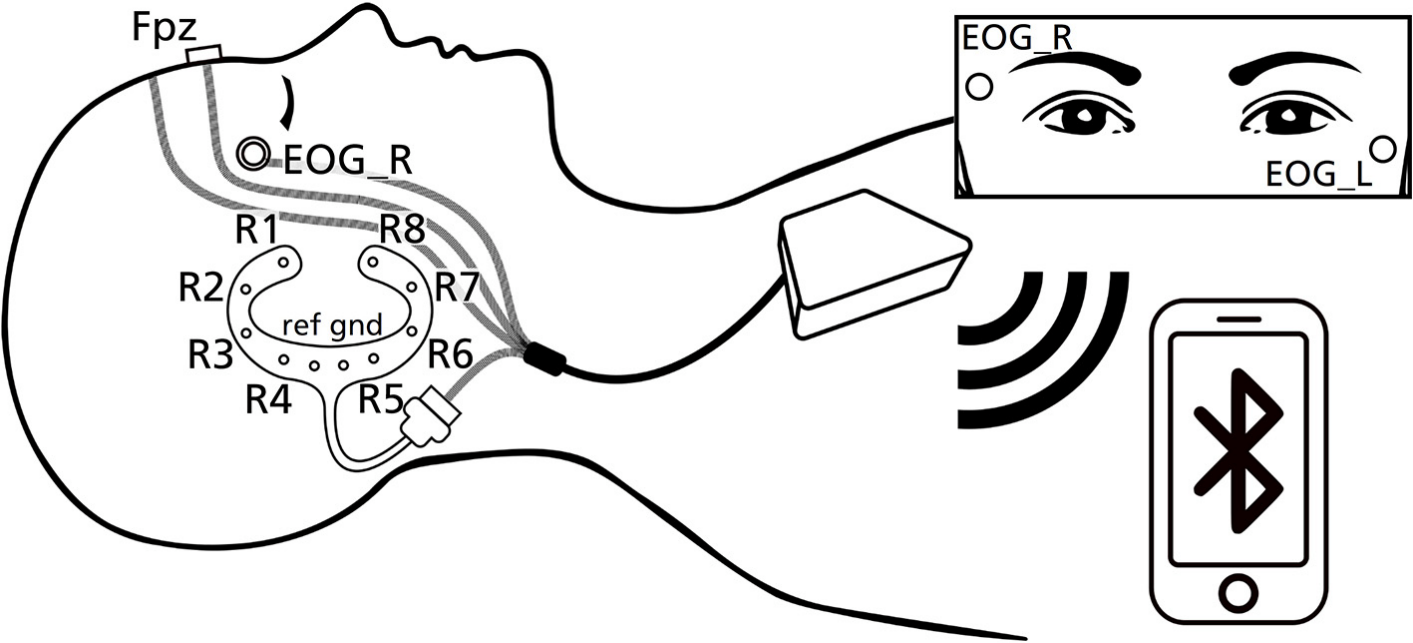
Schematic presentation of the experimental design used in this study, including electrode labels.

The experimenter visited the participants' house in the evening to prepare him or her for the night's recording. Participants were asked to wear their nightwear and no make-up. Participants gave written informed consent to participate in the study before preparation began. The experimental setting is shown in Figure 1. One cEEGrid (21) was prepared with abrasive electrolyte gel (ABRALYT HiCl, Easycap, Germany) and placed around the right ear with a self-adhesive sticker. In addition, a single sintered Ag/AgCl ring electrode was placed on the Fpz location. For the EOG signal, two Ag/AgCl electrodes were placed diagonally near the eyes in accordance with the AASM (American Academy of Sleep Medicine) manual. All ring electrodes were then filled with the electrolyte gel. The electrodes were connected to a SMARTING SLEEP amplifier (mBrainTrain, Serbia), which included a built-in Gyro sensor, and then attached to a chest strap. The amplifier connected via Bluetooth to a commercially available smartphone (Sony Xperia Z1) which was placed close to the bed of the participant. Impedances were checked on the SMARTING app und recording commenced when impedances were generally below 20 kΩ. In order to secure cables for sleep, tubular bandages were applied each to the participant's head and to the connector bundling the cables. Overall, 9 EEG channels (right cEEGrid channels R1-8, Fpz), 2 EOG channels (EOG_L and EOG_R) and 3 Gyro channels were recorded with a sampling rate of 250 Hz for the duration of the night's sleep (reference and ground are located at the center of the cEEGrid and placed on the right mastoid portion, see Figure 1).

### Data Analysis

#### Data Preprocessing

The following preprocessing steps were done to prepare the recorded EEG data for the annotation of the sleep expert scorer and the correlation analysis. For the annotation, three channel layouts (*Fpz*+*EOG, cEEGrid* and *cEEGrid*+*EOG)* were used as listed in Table 1. Channels were selected and if needed re-referenced and relabeled.

**Table 1:** Channel configuration of the three different data sets.

***Data set***	***Fpz+EOG***	***cEEGrid***	***cEEGrid+EOG***
*EEG channels*	Fpz	R1 (R1+R2)/2 - R6 (R2+R3)/2 - (R6+R7)/2 (R3+R4)/2 - R7 -R8	R1 (R1+R2)/2 - R6 (R2+R3)/2 - (R6+R7)/2 (R3+R4)/2 - R7 -R8
*EOG*	EOG_L EOG_R	none	EOG_L - R6 EOG_R - R6

**Figure 2:**
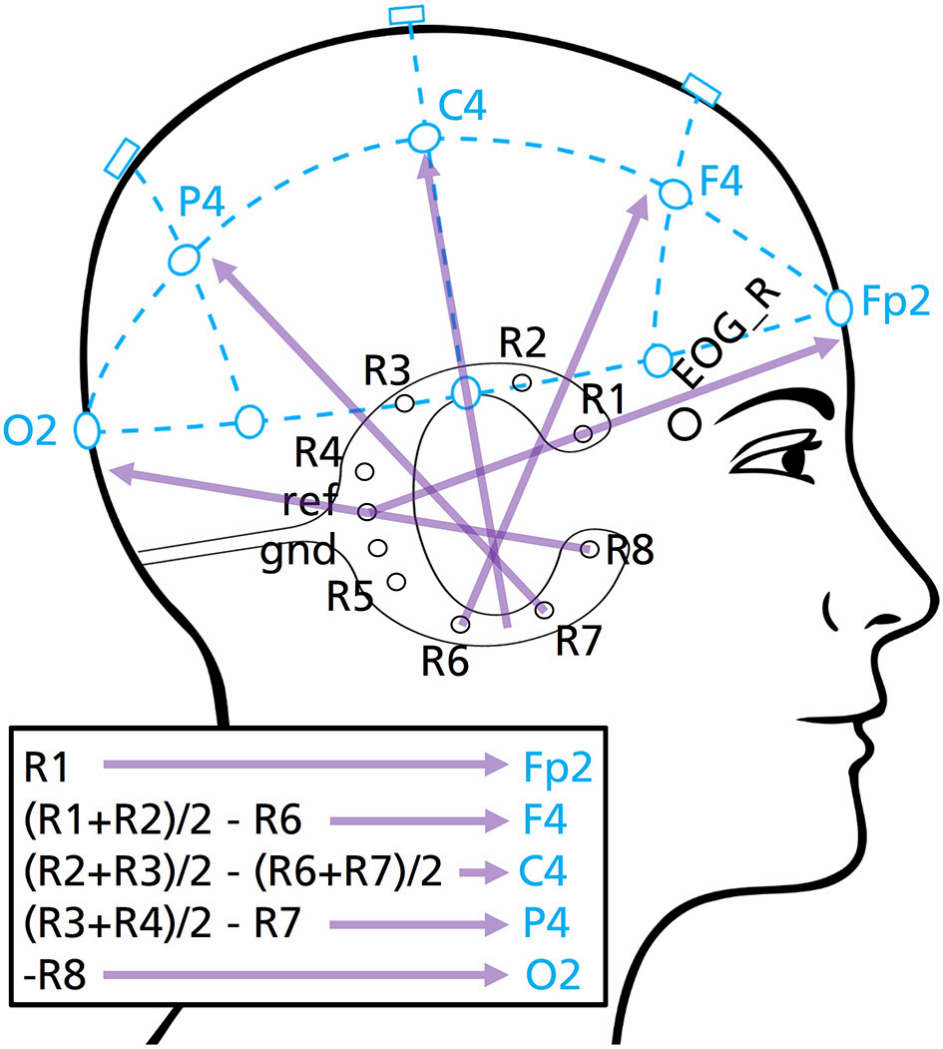
cEEGrid channel combinations used for the *cEEGrid* and *cEEGrid*+*EOG* channel layouts. These bipolar channels extrapolate toward classical PSG scalp positions (Fp2, F4, C4, P4, O2), as illustrated.

The data set *Fpz*+*EOG* is taken as reference with which the first experimental data set *cEEGrid* and the second experimental data set *cEEGrid*+*EOG* is compared with. For the cEEGrid layouts linear combinations of cEEGrid channels were used motivated to approximately represent the EEG measured at classic PSG-relevant scalp positions Fp2, F4, C4, P4 and O2 when referenced to the right mastoid process M2 (as shown in Figure 2). The channel combinations where labeled accordingly to PSG standards to assure their familiarity to the scorer (e.g., Fp2_M2, F4_M2, …). In data set *cEEGrid*+*EOG* two EOG channels are added and re-referenced to R6 as classical mastoid process reference.

After this step, EEG-data was bandpass filtered, using a phase true, 4th order Butterworth filter with a passband of 0.5 to 40 Hz, to reduce electrode drift and noise containing high-frequency components. Following this step, data was down sampled to 125 Hz.

#### Data Annotation of the Sleep Expert

An expert polysomnographic technologist with 14 years of experience in the area of polysomnography (MP, subsequently referred to as expert scorer) annotated the EEG data in consecutive 30 s segments, using an open source, sleep analysis software (26). Sleep staging was done in respect to the AASM guidelines for sleep staging in four annotation conditions described in Table 2 [annotated stages: awake (W), N1, N2, N3 and REM (rapid eye movement)]. Note that the novelty of the setup meant that the technical and digital specifications deviated considerably from AASM guidelines. The sleep staging guidelines, including amplitude thresholds, were observed to the best of the scorer's abilities. In addition, grapho-elements (K-complexes and sleep spindles) were annotated in the condition **Fpz+EOG 2**^**nd**^ **Rating**.

**Table 2:** Description of the four sleep staging annotation conditions.

**Fpz+EOG 1^**st**^ Rating based on data set *Fpz+EOG* of nine participants. Participant number 5 was excluded to inferior data quality based on visual inspection of the experimenter**.
**Fpz+EOG 2**^**nd**^ **Rating** based on data set *Fpz+EOG* of all ten participants. Two years of time in-between 1^st^ and 2^nd^ rating. In addition, grapho-elements (K-complexes and sleep spindles) were annotated.
**cEEGrid Rating** based on data set *cEEGrid* of all ten participants.
**cEEGrid+EOG Rating** based on data set *cEEGrid+EOG* of all ten participants.

#### Correlation Analysis

K-complex and sleep spindle events where extracted by separation of the transient EEG of the cEEGrid channels (R1, …, R8) and Fpz into epochs according to the grapho-element annotations of the expert scorer in **Fpz+EOG 2**^**nd**^ **Rating**. DC-offsets where estimated and reduced by subtracting the average over all samples of the respective epoch. Finally, correlation coefficients were calculated between the reference grapho-elements (annotated epochs in Fpz) and the respective transient data of all possible single channel and bipolar channel combinations of cEEGrid electrodes (36 in total) as shown in the following:

R1,
R1-R2, R2,
R1-R3, R2-R3, R3,
R1-R4, R2-R4, R3-R4, R4,
R1-R5, R2-R5, R3-R5, R4-R5, R5,
R1-R6, R2-R6, R3-R6, R4-R6, R5-R6, R6,
R1-R7, R2-R7, R3-R7, R4-R7, R5-R7, R6-R7, R7,
R1-R8, R2-R8, R3-R8, R4-R8, R5-R8, R6-R8, R7-R8, R8.

To calculate the average correlation coefficient over epochs of one grapho-element or further average over participants, the single correlation coefficients were first Fisher Z transformed than averaged and in a final step back-transformed, by calculating its hyperbolic tangent.

#### Statistical Analysis of Hypnograms

To statistically evaluate the accordance of the different hypnograms, Cohen's Kappa was used to determine the respective inter-rater reliability in three test conditions as described in Table 3 [agreement scale according to (27)]. For a calculation of the reliability over participants, the corresponding hypnograms were concatenated. Therefore, confusion matrices of the different hypnograms were calculated and further used to determine the Cohen's Kappa and its standard error. Cohen's Kappa was calculated for every single sleep stage vs. the rest, and for all sleep stages.

**Table 3:** Description of all statistical test conditions.

**Fpz+EOG test-retest comparing results of Fpz+EOG 1^**st**^ Rating and Fpz+EOG 2^**nd**^ Rating (Participant number 5 not included, since not contained in Fpz+EOG 1^**st**^ Rating)**.
**Fpz+EOG vs. cEEGrid** comparing results of **Fpz+EOG 2**^**nd**^ **Rating** and **cEEGrid Rating**. ^*^
**Fpz+EOG vs. cEEGrid+EOG** comparing results of **Fpz+EOG 2**^**nd**^ **Rating** and **cEEGrid+EOG Rating**. ^*^

* *Only the first 5 h and 20 min of rating results of participant number 1 were used, due to signal loss on several cEEGrid channels during the second half of the night*.

## Results

### Hypnograms

**Figure 3:**
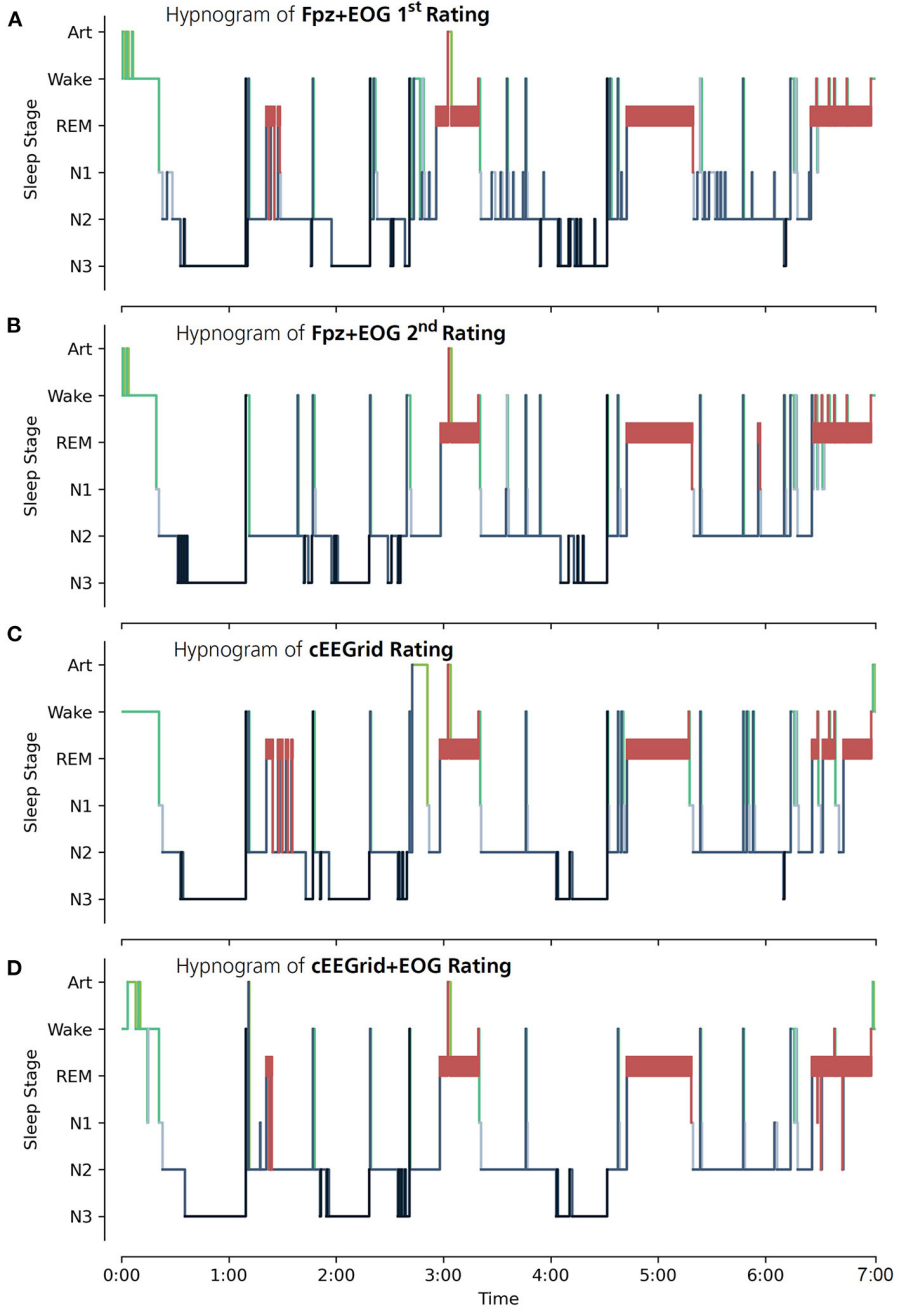
Hypnograms created by the expert scorer are shown for participant number 4. **(A)** Hypnogram of **Fpz+EOG 1**^**st**^ **Rating** using the EEG data of *Fpz*+*EOG*. **(B)** Hypnogram of **Fpz+EOG 2**^**nd**^ **Rating** using the EEG data of *Fpz*+*EOG*. **(C)** Hypnogram of **cEEGrid Rating** using the EEG data of *cEEGrid*. **(D)** Hypnogram of *cEEGrid*+*EOG* Rating using the EEG data of *cEEGrid*+*EOG*. The sleep stages are defined according to the AASM as: Art (Artifact), Wake (Wakefulness), REM (Rapid Eye Movement), N1 (non-REM1), N2 (non-REM2) and N3 (non-REM3).

Hypnograms of all four annotation conditions are shown in Figure 3 for a representative participant. In Figures 3A,B results of **Fpz+EOG 1**^**st**^ **Rating** and **Fpz+EOG 2**^**nd**^ **Rating** are shown, respectively. The results of the corresponding reliability tests **Fpz+EOG test-retest** are listed in the left column of Table 4. Hence the data of participant 5 was not analyzed in **Fpz+EOG 1**^**st**^ **Rating**, the hypnograms of only nine participants were used for this comparison.

**Table 4:** Statistical reliability test results and corresponding standard errors for three test conditions (first: **Fpz+EOG test-retest** comparing results of **Fpz+EOG 1**^**st**^ **Rating** and **Fpz+EOG 2**^**nd**^ **Rating**; second: **Fpz+EOG vs. cEEGrid** comparing results of **Fpz+EOG 2**^**nd**^ **Rating** and **cEEGrid Rating**; third: **Fpz+EOG vs. cEEGrid+EOG** comparing results of **Fpz+EOG 2**^**nd**^ **Rating** and **cEEGrid+EOG Rating**).

	**Fpz+EOG** **test-retest**	**Fpz+EOG vs.** **cEEGrid**	**Fpz+EOG vs.** **cEEGrid+EOG**
W	0.78 ± 0.01	0.71 ± 0.01	0.71 ± 0.01
N1	0.46 ± 0.02	0.37 ± 0.02	0.42 ± 0.02
N2	0.77 ± 0.01	0.62 ± 0.01	0.75 ± 0.01
N3	0.90 ± 0.01	0.85 ± 0.01	0.88 ± 0.01
REM	0.86 ± 0.01	0.69 ± 0.01	0.83 ± 0.01
all	0.78 ± 0.01	0.67 ± 0.01	0.75 ± 0.01

*Combining all participants, the tests results are shown for either every single sleep stage vs. the rest or all sleep stages combined*.

The hypnograms scored an overall substantial agreement with a test-retest reliability of Cohen's κ = 0.78 ± 0.0. The corresponding confusion matrix is shown in Table 5A. W and N2 scored a substantial agreement in the single stage vs. the rest test condition. N1 scored a moderate and N3 and REM an almost perfect agreement. The κ values shown in the left column of Table 4 were taken as representing best possible scores for test conditions **Fpz+EOG vs. cEEGrid** and **Fpz+EOG vs. cEEGrid+EOG**.

**Table 5:** Resulting confusion matrices of (A) condition **Fpz+EOG test-retest** (missing participant number 5, since not included in **Fpz+EOG 1**^**st**^ **Rating**), (B) condition **Fpz+EOG vs. cEEGrid** and (C) condition **Fpz+EOG vs. cEEGrid+EOG**.

**(A) Fpz+EOG test-retest**		***Art***	***W***	***N1***	***N2***	***N3***	***REM***
	*Art*	63	19	1	1	0	5
	*W*	4	927	96	18	2	8
	*N1*	0	58	370	260	0	53
	*N2*	0	117	168	2,870	226	23
	*N3*	1	7	2	39	1,495	0
	*REM*	0	102	91	104	1	1,562
**(B) Fpz+EOG vs. cEEGrid**		*Art*	*W*	*N1*	*N2*	*N3*	*REM*
	*Art*	100	8	2	19	1	2
	*W*	63	769	47	135	10	98
	*N1*	27	86	257	306	3	60
	*N2*	135	71	133	2,958	244	82
	*N3*	2	2	1	198	1,753	1
	*REM*	83	23	70	399	2	1,191
**(C) Fpz+EOG vs cEEGrid+EOG**		*Art*	*W*	*N1*	*N2*	*N3*	*REM*
	*Art*	70	13	21	14	0	14
	*W*	84	714	85	133	3	103
	*N1*	29	51	300	295	1	63
	*N2*	63	11	96	3,288	121	44
	*N3*	6	3	1	241	1,705	1
	*REM*	35	14	75	126	0	1,518

*Each count refers to the annotation of a single 30 second segment. The respective hypnograms of all participants were concatenated to create the confusion matrices. Only the first 5 h and 20 min of rating results of participant number 1 entered the analyses of **(B, C)**, since several cEEGrid electrodes separated from the skin during the second half of the night*.

In Figures 3C,D results **of Fpz+EOG 2**^**nd**^ **Rating** and **cEEGrid Rating** are exemplarily shown for participant number 4. The results of the reliability tests of **Fpz+EOG vs. cEEGrid** and **Fpz+EOG vs. cEEGrid+EOG** are listed in the middle and right column of Table 4 (corresponding confusion matrices are shown in Tables 5B,C). Overall, a substantial agreement is scored in condition **Fpz+EOG vs. cEEGrid** (Cohen's κ = 0.67 ± 0.01) as well as in condition **Fpz+EOG vs. cEEGrid+EOG** (Cohen's κ = 0.75 ± 0.01). A paired-sample *t*-test (one-tailed) of the single participant Cohen's κ values revealed a significant difference in agreement (*p* = 0.006).

In the single stage vs. the rest test condition, N3 scored an almost perfect agreement in both comparisons. Condition REM scored a substantial agreement in **Fpz+EOG vs. cEEGrid** and an almost perfect agreement in **Fpz+EOG vs. cEEGrid+EOG**. Conditions W and N2 scored a substantial agreement in both comparisons. **Fpz+EOG vs. cEEGrid** shows a fair agreement in condition N1 while **Fpz+EOG vs. cEEGrid+EOG** shows a moderate agreement.

### Grapho-Elements

**Figure 4:**
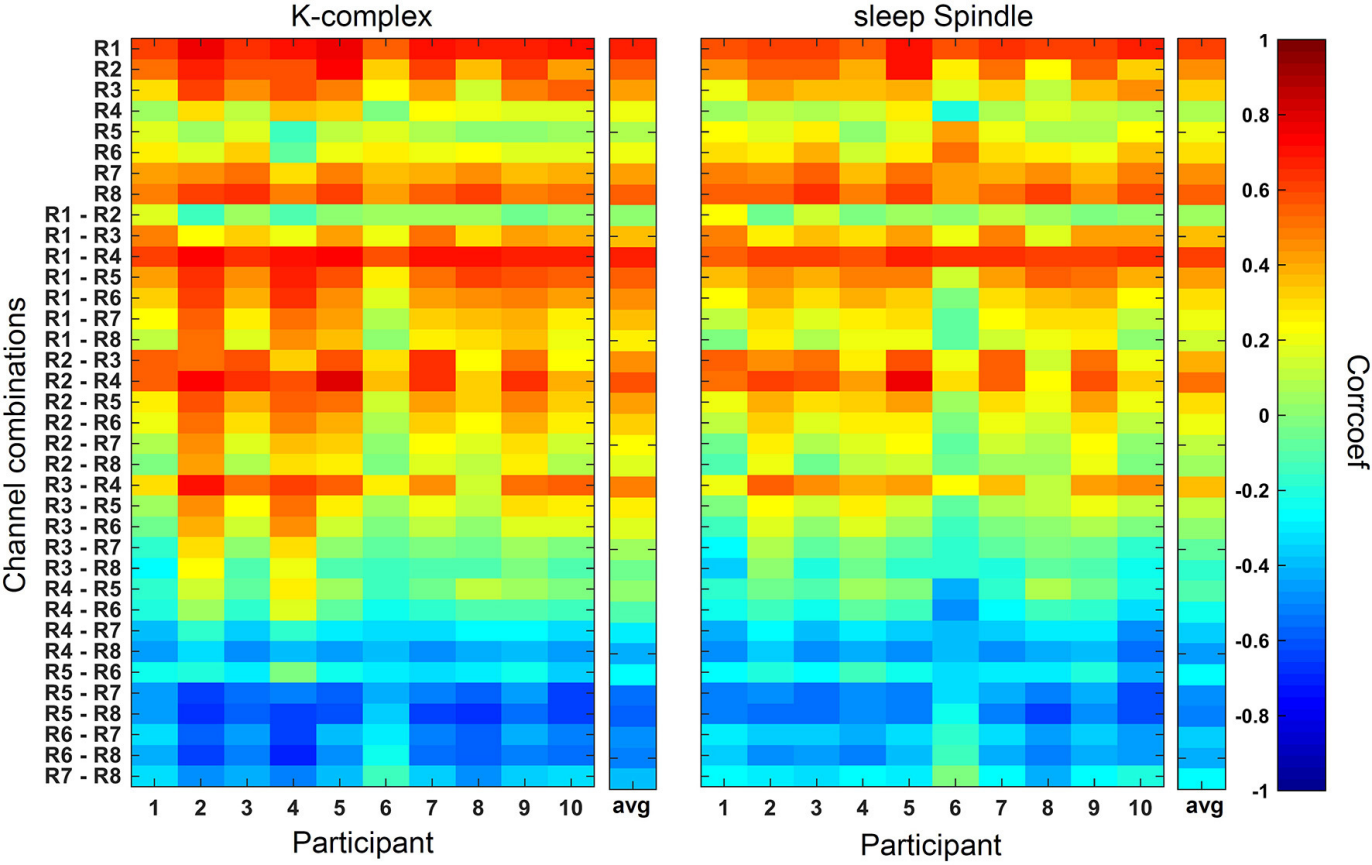
Correlation analysis results averaged over epochs for each single participant and the average over participants (*avg*). Correlation coefficients between grapho-elements (K-complexes and sleep spindles annotated at EEG of Fpz) and the corresponding EEG of every possible single channel and bipolar channel combinations of cEEGrid electrodes are calculated for every epoch, Fisher Z transformed, averaged over epochs (and participants for *avg*), and transformed back.

**Figure 5:**
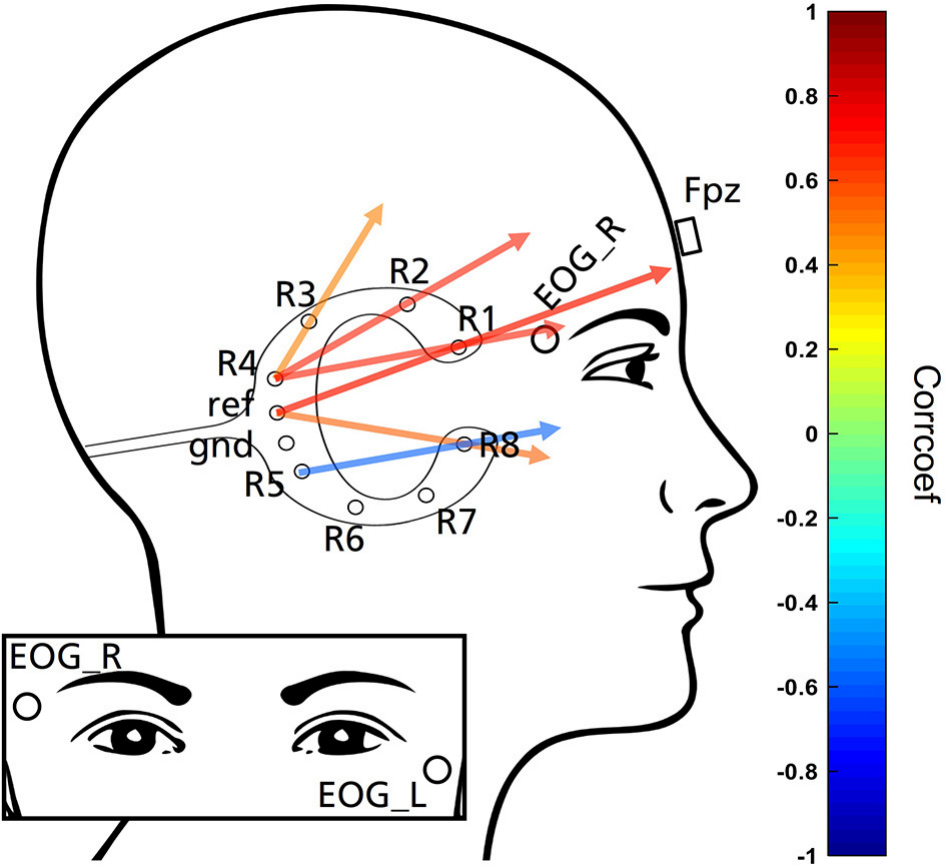
Exemplarily demonstration of six cEEGrid channel combinations. Colors represent the magnitude of correlations. Based on the correlation analysis cEEGrid channel combination that point in the direction of Fpz result in highest absolute correlation and therefore are best suited to represent grapho-elements (K-complexes and sleep spindles) annotated in the EEG recorded at Fpz. Combination R5-R8 pointing in frontal direction due to a negative correlation value.

Figure 4 shows the results of the correlation analysis averaged over epochs for K-complex and sleep spindle events, respectively. The effect is similarly visible in the average over participants and single participant results. R1 and R1-R4 scored the highest positive (K-complex: 0.68, sleep spindle: 0.62) and R5-R8 scored the highest negative average correlation coefficients (K-complex: −0.59, sleep spindle: −0.52). It is noticeable that K-complexes and sleep spindles annotated in EEG recorded at Fpz are best represented in EEG of cEEGrid channel combinations that point in the direction of Fpz. This directional dependence is exemplarily shown in Figure 5 for six cEEGrid channel combinations. Overall, these results confirm the assumption that combinations of ear EEG channels can be used to estimate the EEG measured at further distanced location of the scalp and therefore the selection of channel combinations used for *cEEGrid* and *cEEGrid*+*EOG*, cf. Table 1.

## Discussion

The present study explored whether flex-printed ear-EEG sensors can be used to capture sleep stages from recordings performed with a wireless amplifier and off-the-shelf smartphone technology at home. The correlation analysis approach provides evidence that combinations of ear EEG channels can be used to extrapolate information measured at traditional locations on the scalp, like Fpz. Overall, the results support the selection of those cEEGrid channel combinations that were used for the *cEEGrid* and *cEEGrid*+*EOG* analysis. Here, cEEGrid channels were combined and re-named to mimic EEG signals that may be recorded from traditional PSG scalp electrodes Fp2, F4, C4, P4 and O2. The quality of the hypnograms in comparison to the *Fpz*+*EOG* hypnograms differed depending on the inclusion of additional EOG channels in the analysis. The hypnograms based on *cEEGrid* only showed significantly less agreement than the *cEEGrid*+*EOG* hypnograms. A comparison of Cohen's κ values of the columns of Table 4 shows that **Fpz+EOG vs. cEEGrid+EOG** scored comparable results to the test-retest reliability of the expert scorer. It is further noticeable that even sleep stage N1 showed a comparable reliability. An investigation of the inter-rater reliability of hypnograms created according to the AASM for seven different trained sleep scorers yielded comparable values [N1: Cohen's κ = 0.46, REM: Cohen's κ = 0.91; (28)]. Therefore, while the reliability of scores appears low for some sleep stages, it is not lower than what is typical for manually scored hypnograms. We expect that automated scoring may outperform manual scorers and yield better results in the near future (29). The results of the correlation analysis further indicate that annotation of grapho-elements, like K-complexes and sleep spindles, should be possible in ear-EEG data.

In the current study, we found that off-the-shelf smartphone technology, when combined with a wireless amplifier and a potentially easy-to-administer ear-EEG electrodes array, may be sufficient to provide hypnograms from home sleep data. Note however that ear-EEG performed better in combination with additional EOG electrodes than by itself, in particular when determining REM sleep. REM sleep can be challenging to differentiate from wake phases without EOG which provides essential information on the vertical and horizontal eye movements that signify REM sleep. Similarly, slow rolling eye movements clearly visible in the EOG channels may indicate the transition from wake to N1 sleep. Due to the importance of the EOG information for sleep scoring, it would be interesting for future studies to test if a scoring using EOG alone is possible (24) showed only moderate agreement for all sleep stages combined (κ = 0.42 ± 0.21) between hypnograms based on cEEGrid vs. hypnograms based on full PSG and attributed the absence of an EOG as a possible reason for the discrepancy. The current study is based on a manual scoring of the hypnograms. The use of channel combinations approximating the signal of classical PSG channels combined with suitable channel labels, instead of direct cEEGrid channels, could also be one reason for a better agreement, as supposed in (25). In the current study, κ scores for N1 sleep ranged from 0.37 to 0.46 across all conditions, mirroring the often noted difficulty to identify the transition from wake state to N1 sleep even in fully equipped PSG settings (28).

The strength of this study – collecting data at home while participants sleep in their beds – also depicts its limits. By using miniaturized, portable equipment, the comparison of the new system was not to a full PSG but to an approximation of it using just a single frontal electrode at Fpz in addition to the EOG electrodes. The minimal setup according to the AASM to determine sleep stages demands three scalp electrodes at positions F4, C4 and O2, referenced to the mastoid (30). The Fpz position was chosen based on studies showing sleep staging to work well-when based on a single channel forehead recording (17, 18). In addition, we aimed for EEG positions on hairless skin. In future studies, a validation of a re-designed position layout against the gold standard of sleep laboratory PSG will be needed to give a better representation of its accuracy. In the current study, we used the SMARTING amplifier without utilizing the built-in gyroscope. For future applications, movement sensors such as accelerometers and gyroscopes included in the hardware may offer additional information for sleep analysis. It would be worthwhile to track body positions and reconstruct breathing patterns from chest movements, without adding to the bulkiness of additional sensors.

Of the relevant characteristics that expert sleep scorer require, eye movements and grapho-elements are of great importance. They will likely continue to play a prominent role in defining hypnograms as the field moves toward automatic sleep stage detection (31). Concerning eye movements, the cEEGrid proved a useful approximation to relevant scalp positions but it was not able to provide sufficient EOG information, at least when used on a single side only. To improve on the design, extending the electrode array to include near-eye positions, or possibly emulating EOG by using two cEEGrids (one on each ear) and cross-referencing between the two may be advantageous (22). A recent adaptation of the cEEGrid to flex-printed forehead EEG delivered high-quality EEG signals of forehead and facial positions with minimal discomfort or inconvenience over the course of 8 h (32). Encompassing standard EOG positions in the grid, this electrode array may provide a signal suitable for detection of REM, though it has not been tested for wear during sleep. As noted previously (21), it is only available in one size, which can make fitting difficult, especially in the elderly who tend to have larger ears. The cEEGrid is not flexible and adjustable enough to be worn comfortably by everyone. Ideally, ear-EEG electrode grids should be manufactured from a more flexible material and be available in different sizes to allow easier self-application.

Concerning grapho-elements, we found that sleep spindles and K-complexes are best represented in cEEGrid channel combinations that point in the direction of Fpz in a data-driven approach. In a future study, we plan to directly compare ear-EEG channel combinations with EEG measured at several scalp positions to further validate ear-EEG solutions for sleep EEG acquisition. Recently, suggestions have been made for the source of sleep spindle and K-complex generators (33, 34) that may facilitate an ideal layout of bipolar channels on the hairless skin to best capture sleep characteristics. In the future, a generator-driven approach may be helpful to place bipolar channels in a way that ideally capture the source of sleep stage characteristics like sleep spindles and K-complexes. Concerning ear-EEG, the source-sensor relationship has recently been evaluated by simulations to compare cEEGrid ear-EEG with 128-channel cap-EEG (23). Using the same forward modeling approach, a new arrangement of electrodes, i.e., oriented toward a K-complex generator, may be compared to full scalp EEG to provide an estimate of sensitivity to the regions in question. This approach may help in finding the best trade-off between comfortable, unobtrusive sensors and data quality. Further miniaturization and optimization of wireless EEG systems is an additional point to be addressed in future. Current mobile EEG systems already include movement sensors. Depending on the placement of the amplifier on the body during night, movement sensors could be used to get additional information, such as body position and breathing patterns.

The AASM criteria form a helpful standard that has been refined over decades. However, as the idea of sleep monitoring moves from hand-coding by trained personal to automatic decoding with machine learning methods, inflexible procedures of the PSG may constrain the development of new standards for identifying sleep stage characteristics. Disruptive technologies may be needed to help identify normal and abnormal events during sleep in ecologically valid settings. Ear-EEG acquisition seems suitable for the development of comfortable, discrete and robust sleep EEG system that work at home, can be self-administered and are unobtrusive during wear.

## Data Availability Statement

The datasets presented in this article are not readily available because sharing of raw data was not included in the ethics statement. Requests to access the datasets should be directed to Carlos F. da Silva Souto, carlos.filipe.da.silva.souto@idmt.fraunhofer.de.

## Ethics Statement

The studies involving human participants were reviewed and approved by the Research Impact Assessment and Ethics Committee at the Carl von Ossietzky University of Oldenburg. The participants provided their written informed consent to participate in this study.

## Author Contributions

IM and SD designed the experiment. IM collected the data. IM and MB analyzed all 2018 data. MP scored all sleep data and coded grapho-element annotations. CS analyzed all 2020 data devised executed the correlations, and grapho-element analysis. WP, CS, and KIW wrote the manuscript. SD, IM, MB, and MP offered feedback during the writing process. All authors contributed to the article and approved the submitted version.

## Conflict of Interest

MP was commissioned to score sleep data independently in 2018 by SD and in 2020 by KIW. The remaining authors declare that the research was conducted in the absence of any commercial or financial relationships that could be construed as a potential conflict of interest.

## References

[1] Mayer G Fietze I Fischer J Penzel T Riemann D Rodenbeck A. S3 Leitlinie Nicht erholsamer Schlaf. Somnologie 13(Supplement1):1–160.

[2] Feige B Al-Shajlawi ANAM Nissen C Voderholzer U Hornyak M Spiegelhalder. Does REM sleep contribute to subjective wake time in primary insomnia? A comparison of polysomnographic and subjective sleep in 100 patients. J Sleep Res. (2008) 17:180–90. 10.1111/j.1365-2869.2008.00651.x 18482106

[3] Russo K Goparaju B Bianchi MT. Consumer sleep monitors: is there a baby in the bathwater? Nat Sci Sleep. (2015) 7:147. 10.2147/NSS.S94182 26604847 PMC4640400

[4] Ko PRT Kientz JA Choe EK Kay M Landis CA Watson NF. Consumer sleep technologies: a review of the landscape. J Clin Sleep Med. (2015) 11:1455–61. 10.5664/jcsm.5288 26156958 PMC4661339

[5] Gavriloff D Sheaves B Juss A Espie CA Miller CB Kyle SD. Sham sleep feedback delivered via actigraphy biases daytime symptom reports in people with insomnia: Implications for insomnia disorder and wearable devices. J Sleep Res. (2018) 27:e12726. 29989248 10.1111/jsr.12726

[6] Miettinen T Myllymaa K Westeren-Punnonen S Ahlberg J Hukkanen T Toyras J. Success rate and technical quality of home polysomnography with self-applicable electrode set in subjects with possible sleep bruxism. IEEE J Biomed Health Inform. (2018) 22:1124–32. 10.1109/JBHI.2017.2741522 28829322

[7] Myllymaa S Muraja-Murro A Westeren-Punnonen S Hukkanen T Lappalainen R Mervaala E. Assessment of the suitability of using a forehead EEG electrode set and chin EMG electrodes for sleep staging in polysomnography. J Sleep Res. (2016) 25:636–45. 10.1111/jsr.12425 27230805

[8] Shustak S Inzelberg L Steinberg S Rand D Pur MD Hillel I. Home monitoring of sleep with a temporary-tattoo EEG, EOG and EMG electrode array: a feasibility study. J Neural Eng. (2019) 16:026024. 10.1088/1741-2552/aafa05 30566912

[9] Ferster ML Lustenberger C Karlen W. Configurable mobile system for autonomous high-quality sleep monitoring and closed-loop acoustic stimulation. IEEE Sensors Lett. (2019) 3:1–4. 10.1109/LSENS.2019.2914425

[10] Mikkelsen KB Villadsen DB Otto M Kidmose P. Automatic sleep staging using ear-EEG. BioMed Eng OnLine. (2017) 16:111. 10.1186/s12938-017-0400-5 28927417 PMC5606130

[11] Mikkelsen KB Ebajemito JK Bonmati-Carrion MA Santhi N Revell VL Atzori G. Machine-learning-derived sleep–wake staging from around-the-ear electroencephalogram outperforms manual scoring and actigraphy. J Sleep Res. (2019) 28:e12786. 10.1111/jsr.12786 30421469 PMC6446944

[12] Nakamura T Alqurashi YD Morrell MJ Mandic DP. Hearables: automatic overnight sleep monitoring with standardized in-ear EEG sensor. IEEE Trans Biomed Eng. (2019) 67:203–12. 10.1109/TBME.2019.2911423 31021747

[13] Younes M Soiferman M Thompson W Giannouli E. Performance of a new portable wireless sleep monitor. J Clin Sleep Med. (2017) 13:245–58. 10.5664/jcsm.6456 27784419 PMC5263080

[14] Levendowski DJ Ferini-Strambi L Gamaldo C Cetel M Rosenberg R Westbrook PR. The accuracy, night-to-night variability, and stability of frontopolar sleep electroencephalography biomarkers. J Clin Sleep Med. (2017) 13:791–803. 10.5664/jcsm.6618 28454598 PMC5443740

[15] Debellemaniere E Chambon S Pinaud C Thorey V Dehaene D Léger D. Performance of an ambulatory dry-EEG device for auditory closed-loop stimulation of sleep slow oscillations in the home environment. Front Hum Neurosci. (2018) 12:14. 10.3389/fnhum.2018.00088 29568267 PMC5853451

[16] Arnal PJ Thorey V Debellemaniere E Ballard ME Bou Hernandez A Guillot A. The dreem headband compared to polysomnography for electroencephalographic signal acquisition and sleep staging. Sleep. (2020) 43:1–13. 10.1093/sleep/zsaa097 32433768 PMC7751170

[17] Lucey BP Mcleland JS Toedebusch CD Boyd J Morris JC Landsness EC. Comparison of a single-channel EEG sleep study to polysomnography. J Sleep Res. (2016) 25:625–35. 10.1111/jsr.12417 27252090 PMC5135638

[18] Popovic D Khoo M Westbrook P. Automatic scoring of sleep stages and cortical arousals using two electrodes on the forehead: validation in healthy adults. J Sleep Res. (2014) 23:211–21. 10.1111/jsr.12105 24313630 PMC4156826

[19] Howe-Patterson M Pourbabaee B Benard F. Automated detection of sleep arousals from polysomnography data using a dense convolutional neural network. In: 2018 Computing in Cardiology Conference (CinC). IEEE (2018). 10.22489/CinC.2018.232

[20] Pourbabaee B Patterson MH Patterson MR Benard F. SleepNet: automated sleep analysis via dense convolutional neural network using physiological time series. Physiol Meas. (2019) 40:084005. 10.1088/1361-6579/ab3632 31349239

[21] Debener S Emkes R Vos MD Bleichner M. Unobtrusive ambulatory EEG using a smartphone and flexible printed electrodes around the ear. Sci Rep. (2015) 5:1–11. 10.1038/srep16743 26572314 PMC4648079

[22] Bleichner MG Debener S. Concealed, unobtrusive ear-centered EEG acquisition: cEEGrids for transparent EEG. Front Hum Neurosci. (2017) 11:163. 10.3389/fnhum.2017.00163 28439233 PMC5383730

[23] Meiser A Tadel F Debener S Bleichner MG. The sensitivity of ear-EEG: evaluating the source-sensor relationship using forward modeling. Brain Topog. (2020) 33:665–76. 10.1007/s10548-020-00793-2 32833181 PMC7593286

[24] Sterr A Ebajemito JK Mikkelsen KB Bonmati-Carrion MA Santhi N Della Monica C. Sleep EEG derived from behind-the-ear electrodes (cEEGrid) compared to standard polysomnography: a proof of concept study. Front Hum Neurosci. (2018) 12:452. 10.3389/fnhum.2018.00452 30534063 PMC6276915

[25] Mikkelsen KB Tabar YR Kappel SL Christensen CB Toft HO Hemmsen MC. Accurate whole-night sleep monitoring with dry-contact ear-EEG. Sci Rep. (2019) 9:81–7. 10.1038/s41598-019-53115-3 31727953 PMC6856384

[26] Combrisson E Vallat R Eichenlaub JB O'Reilly C Lajnef T Guillot A. Sleep: an open-source python software for visualization, analysis, and staging of sleep data. Front Neuro. (2017) 11:60. 10.3389/fninf.2017.00060 28983246 PMC5613192

[27] Landis JR Koch GG. The measurement of observer agreement for categorical data. Biometrics. (1977) 33:159–74. 10.2307/2529310 843571

[28] Danker-Hopfe H Anderer P Zeitlhofer J Boeck M Dorn H Gruber G. Interrater reliability for sleep scoring according to the Rechtschaffen & Kales and the new AASM standard. J Sleep Res. (2009) 18:74–84. 10.1111/j.1365-2869.2008.00700.x 19250176

[29] Malafeev A Laptev D Bauer S Omlin X Wierzbicka A Wichniak A. Automatic human sleep stage scoring using deep neural networks. Front Neurosci. (2018) 12:781. 10.3389/fnins.2018.00781 30459544 PMC6232272

[30] Berry RB Brooks R Gamaldo CE. The AASM Manual For the Scoring of Sleep and Associated Events: Rules, Terminology and Technical Specifications, version 2.6. 0. Darien: American Academy of Sleep Medicine (2020).

[31] Aboalayon KAI Faezipour M Almuhammadi WS Moslehpour S. Sleep stage classification using EEG signal analysis: a comprehensive survey and new investigation. Entropy. (2016) 18:272. 10.3390/e18090272

[32] Blum S Emkes R Minow F Anlauff J Finke A Debener S. Flex-printed forehead EEG sensors (fEEGrid) for long-term EEG acquisition. J Neural Eng. (2020) 17:1–15. 10.1088/1741-2552/ab914c 32380486

[33] Ioannides AA Liu L Kostopoulos GK. The Emergence of Spindles and K-complexes and the role of the dorsal caudal part of the anterior cingulate as the generator of K-complexes. Front Neurosci. (2019) 13:814. 10.3389/fnins.2019.00814 31447635 PMC6692490

[34] Latreille V von Ellenrieder N Peter-Derex L Dubeau F Gotman J Frauscher B. The human K-complex: insights from combined scalp-intracranial EEG recordings. Neuroimage. (2020) 213:116748. 10.1016/j.neuroimage.2020.116748 32194281

